# Expression Patterns of GATA3 in Classical Hodgkin Lymphoma: A Clinico-Pathological Study

**DOI:** 10.3390/diseases12030051

**Published:** 2024-02-29

**Authors:** Alexandra Papoudou-Bai, Epameinondas Koumpis, Georgia Karpathiou, Eleftheria Hatzimichael, Panagiotis Kanavaros

**Affiliations:** 1 Department of Pathology, Faculty of Medicine, School of Health Sciences, University of Ioannina, 45500 Ioannina, Greece; apapoudou@uoi.gr; 2 Department of Hematology, Faculty of Medicine, School of Health Sciences, University of Ioannina, 45500 Ioannina, Greece; an.koumpis@uoi.gr; 3 Department of Pathology, University Hospital of Saint-Etienne, 42100 Saint-Etienne, France; georgia.karpathiou@chu-st-etienne.fr; 4 Department of Anatomy-Histology-Embryology, Faculty of Medicine, School of Health Sciences, University of Ioannina, 45500 Ioannina, Greece; pkanavar@uoi.gr

**Keywords:** Hodgkin lymphoma, GATA3, immunohistochemistry, prognosis

## Abstract

GATA3 is a transcription factor involved in T-cell maturation and has been previously shown to be aberrantly overexpressed in malignant Hodgkin and Reed–Sternberg (HRS) cells of classical Hodgkin lymphoma (cHL). However, the immunophenotypes of the cell types expressing GATA3 have not been precisely characterized so far in cHL tissues. In this single-center retrospective cohort study we analyzed the expression patterns of GATA3 alone and in combination with B, T, NK or macrophage-associated markers in 73 cases with newly diagnosed cHL and investigated for a possible correlation with clinical and laboratory parameters. Immunohistochemistry (single and double) was performed using GATA3 alone and in combination with CD20, CD3, CD56, CD68, CD30 or CD15. Clinical and laboratory parameters were collected and correlated with the expression of GATA 3. GATA3 nuclear expression was found in HRS cells in 39/73 (54%) cases of cHL. The Nodular Sclerosis (NS) subtype showed the highest positivity rate (35/56, 63%), followed by mixed cellularity (MC; 4/14, 29%) and lymphocyte rich (LR; 0/3). Double immunostainings showed that GATA3 was expressed by CD30+ or CD15+ HRS cells and a few CD3+ T-cells, whereas GATA3 expression was not detected in CD20, CD56 or CD68+ cells. GATA3-negative cHL was significantly associated with unfavorable prognostic factors such as older age at diagnosis and increased levels of serum β2-microglobulin. The heterogenous expression patterns of GATA3 in HRS cells that were observed in a substantial proportion of cHL, mainly in the NS subtype, further support the biological heterogeneity of cHL.

## 1. Introduction

Hodgkin lymphoma (HL) represents approximately 10% of all lymphomas in the Western world [1,2,3]. HL is classified as classical Hodgkin lymphoma (cHL) and as nodular lymphocyte predominant HL (NLPHL) according to the fifth edition of the World Health Organization Classification of Haematolymphoid Tumors [4]. According to the International Consensus Classification of Mature Lymphoid Neoplasms, NLPHL belongs to the group of Mature B-cell neoplasms and is classified as nodular lymphocyte predominant B-cell lymphoma [5]. cHL is characterized histologically by the presence of rare malignant cells, the Hodgkin and Reed–Sternberg (HRS) cells, which are immersed in an abundant reactive microenvironment [1,2,3,6]. The HRS cells are derived from germinal center (GC) B-cells that are characterized by large loss of the typical B-cell gene expression program [3]. Although the mechanisms responsible for the downregulation of the B-cell expression program in HRS cells need further studies, some contributing factors were identified [3]. These factors include upregulation of transcription factors that suppress the B-cell gene expression program (e.g., NOTCH1, ID2, ABF1, STAT5), downregulation of transcription factors for genes involved in the B-cell differentiation program (e.g., BOB1, OCT2, PU.1), as well as epigenetic silencing of B-cell genes [3].

In a fraction of cHL (30–40%), HRS cells harbor the Epstein–Barr virus (EBV), which has been implicated in the pathogenesis of EBV-positive tumors [1,2,3,7]. There is evidence that EBV plays an important role in the rescue of crippled GC B-cells from apoptotic cell death and hence the virus is considered as a major player in the early steps of pathogenesis of EBV-positive cHL cases by participating in the transformation process to malignant HRS cells [3].

New methods in molecular biology have allowed for important insights into the pathobiology of cHL. The study of HRS cells by molecular biology methods revealed significant roles of genetic lesions affecting members of the Janus kinase/signal transducers and activators of transcription (JAK/STAT) and the nuclear factor-κB (NF-κB) cellular signaling pathways, as well as factors that affect immune escape of HRS cells [1,2,3]. An interplay of such genetic lesions and multiple interactions of HRS cells with cell populations belonging to the reactive tumor microenvironment leads to the constitutive activation of several cellular signaling pathways that support survival and proliferation of HRS cells [1,2,3].

cHL is a potentially curable disease and most patients experience long term remission with first-line treatment. However, approximately 20% of them will relapse, while 10–15% are primary refractory [1,2,3]. Novel therapeutic agents, such as programmed cell death-1 (PD-1) inhibitors, alone or in combination with chemotherapy, have shown to improve the outcomes of patients with relapse [1] and most recently in advanced disease in the front-line setting [8].

GATA binding protein 3 (GATA3) is a member of a family of transcription factors comprising six members that identify sequences containing G-A-T-A in the target gene and bind to the DNA target via 2 zinc-finger domains [9]. The members of the GATA family are divided into two groups: (a) the first group consists of GATA1, GATA2, and GATA3 proteins which mainly participate in the development of nervous and hematopoietic systems, and (b) the second group includes GATA4, GATA5, and GATA6 proteins which are associated with the development of endodermal and mesodermal organs [9]. GATA3 is essential for T-cell development and is involved in the regulation of T helper-2 (Th2) differentiation by affecting the genes implicated in Th2 cytokine production [9]. 

As far as T-cell malignancies are concerned, molecular alteration of GATA3 has been associated with early T-cell precursor acute lymphoblastic leukemia, and immunostochemical expression of GATA3 protein in peripheral T-cell lymphoma (ranging from 20 to 40%) has been linked to reduced overall survival [9].

Most importantly, a recent study showed that GATA3 is a bona fide proto-oncogene in T-cell lymphoproliferative neoplasms across the spectrum of immature to mature T-cell leukemias/lymphomas [10]. In this study, Geng et al. used cell lines, mouse models, and histological specimens from T-cell malignancies [peripheral T-cell lymphomas—not otherwise specified (NOS), cutaneous T-cell lymphoma (CTCL), and T-cell acute lymphoblastic leukemia (T-ALL)] and provided evidence that GATA3 can induce transcriptional programs which promote T-cellular growth and proliferation [10]. Moreover, they noticed that some selected GATA3 gene targets such as the p300/GATA3 complex and GATA3 acetylation may be novel therapeutic targets for more effective treatments for these aggressive GATA-3 driven T-cell malignant neoplasms [10]. 

Interestingly, there is evidence of GATA3 aberrant overexpression in HRS cells in cHL [11,12,13,14,15]. Kuppers et al. demonstrated using large-scale microarrays that GATA3 was aberrantly overexpressed in cHL cells (in B-cell derived HL lines L428, L1236, and KMH2) while this was not the case in mature B-cells and several other B-cell lymphomas [11]. Later, Stanelle et al. showed that mechanisms leading to overexpression of GATA3 in cHL involve deregulation of Notch-1 and NF-κB signaling pathways [12]. Consequently, GATA-3 overexpression contributes to the regulation of multiple cytokines and activators (IL-5, IL-13 and STAT4) [12]. In support of this concept, Stanelle et al. showed by immunohistochemistry a significant positive correlation between GATA3 and IL-13 expression in HRS cells in cHL tissues [12]. They analyzed 16 cases of cHL with evaluable staining for both GATA3 and IL-13 proteins and found that (a) all 16 cases exhibited at least 60% immunohistochemical positivity for IL-13 in HRS cells and (b) the cases displaying elevated IL-13 positivity in 80%-90% in HRS cells also showed increased GATA3 positivity in the majority of HRS cells [12]. Based on these results, Stanelle et al. suggested that GATA3 may influence gene expression in HRS cells and their interaction with other cells in the microenvironment, thus shaping tumor pathophysiology [12]. Recently, Kezlarian et al. explored the use of GATA3 immunohistochemical analysis in the differential diagnosis between cHL and other mimicking pathological entities [14]. They reported that a. GATA3 was positive in 34/49 (80%) of cHLs, b. the Nodular Sclerosis (NS) subtype showed the highest positivity rate (23/26; 87%), followed by mixed cellularity (MC; 9/13, 70%), and lymphocyte rich (LR; 2/3, 67%), and c. GATA3 was positive in 3/4 primary mediastinal large B-cell lymphomas (PMBL) [14]. In contrast, GATA3 was not detected in any NLPHL, Epstein–Barr virus (EBV) positive large B-cell lymphoma (LBCL) (EBV+ LBCL), T-cell/histiocyte-rich large B-cell lymphoma (TCHRBCL), and diffuse large B-cell lymphoma (DLBCL) cases [14]. In line with the immunohistochemical results of Kezlarian et al., Hyun-Jung Kim et al. showed that GATA3 was expressed in the majority of cHL cases (10/13, 76%) [15] and Atayar et al. found GATA3 expression in 8/12 cHL cases (67%) and in 0/7 NLPHL cases [13]. Moreover, Atayar et al. confirmed their immunohistochemical results in tissues by showing GATA-3 protein expression using immunocytochemistry only in the three cHL cell lines but not in the one NLPHL cell line [13].

Prompted by the above studies, and since the immunophenotypes of the cell types expressing GATA3 have not been precisely characterized so far in cHL tissues, a. we analyzed the immunohistological expression patterns of GATA3 in 73 cHL cases, b. we performed double immunostaining using GATA3 in combination with CD20, CD3, CD56, CD68, CD30 or CD15 in order to provide detailed immunohistological information concerning the cell types expressing GATA3 in cHL, and c. we investigated whether there is any correlation between the immunohistological expression patterns of GATA3 and clinical and laboratory data such as disease status, treatment response, clinical outcome, and various laboratory parameters. Furthermore, since GATA3 has not been analyzed so far in relation to the presence of EBV in cHL tissues and in view of the in vitro findings that GATA-3 was induced in most EBV-infected GC-lymphoblastoid cell lines (LCLs) [16], we also studied GATA3 expression in relation to the EBV status.

## 2. Materials and Methods

### 2.1. Materials

This was a single-center retrospective cohort study that included 73 consecutive patients with newly diagnosed cHL (56 NS and 14 MC and 3 LR) treated at the Department of Hematology, University Hospital of Ioannina, Greece, between 1999 and 2019. Moreover, one case with breast carcinoma tissue (positive control), four cases of NLPH, which were all GATA3 negative in a previous study (negative control) [14], and one case of reactive lymph node and one case of a tonsil with reactive lymphoid tissue were included in the study. The characteristics of the patients and the clinical data, including the disease status, laboratory values, treatment response, and clinical outcome were collected and recorded. Tissue samples were retrieved from the archives of the Department of Pathology, University Hospital of Ioannina, Greece. The study was approved by the Research Committee of the University General Hospital of Ioannina [Approval reference: 16/9-5-2019 (number 17)]. 

### 2.2. Methods

Immunohistochemical studies were performed in formalin-fixed paraffin embedded tumor tissue sections (4 μm thick) using an automated immunostaining system (OMNIS, Dako-Agilent, Santa Clara, CA, USA) [17]. The following antibodies were used: GATA3: clone L50-823, mouse monoclonal, ZETA corporation, 1:100; CD20: clone L26, mouse monoclonal, Abcam, 1:100; CD3: clone PS1, mouse monoclonal, Novocastra/Leica Biosystems, 1:200; CD68: clone KP-1, mouse monoclonal, Abcam, 1:3000; CD56: clone 123C3, mouse monoclonal Dako, 1:50; CD30: clone Ber-H2, mouse monoclonal, Dako, 1:30; and CD15: clone MMA (LeuM1), mouse monoclonal, Diagnostic BioSystems, 1:150. Negative controls in which the tissue was incubated with only the antibody diluent, without the primary antibody, were used in our experiments. Double immunohistochemical staining for concomitant detection of GATA3, CD20, CD3, CD56, CD68, CD30 or CD15 was performed as previously described using the double-streptavidin-biotin peroxidase-labeled (LSAB)/alkaline phosphatase/anti-alkaline phosphatase (APAAP) immunohistochemical procedures [18]. EBV was detected using EBER1/2 in situ hybridization according to standard protocol [19].

### 2.3. Statistical Analysis

Clinical and laboratory parameters were collected and correlated with the expression of GATA 3 in patients with cHL. For the statistical analysis, χ^2^ test and *t*-test or Mann–Whitney U test were used to compare categorical and continuous variables in GATA3 positive and GATA3 negative patients, respectively. 

Overall survival (OS) was defined as the time from treatment initiation to death from any cause, or last follow-up. The Kaplan–Meier method was used to calculate OS. Survival curves were compared using the log-rank test. Parameters with *p*-values < 0.10 along with GATA3 expression were evaluated in a multivariate analysis using Cox’s proportional hazard model. *p* values < 0.05 were considered statistically significant. Statistical analysis was performed using the SPSS 28 program (SPSS, Chicago, IL, USA).

## 3. Results

### 3.1. GATA3 Expression Patterns

**Figure 1:**
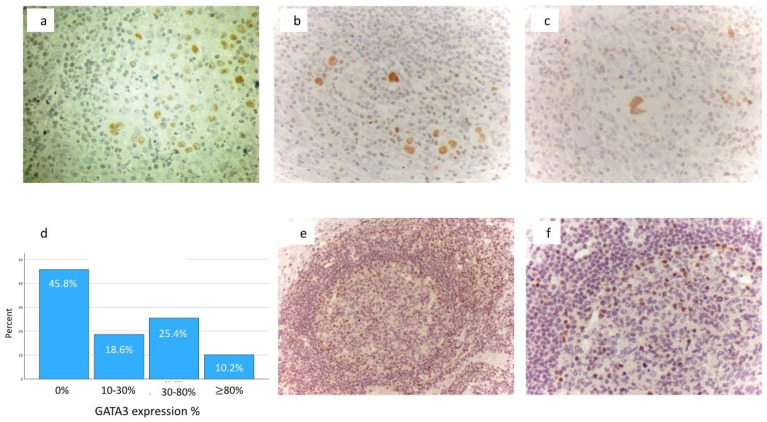
(**a**–**c**): Nuclear immunohistochemical expression of GATA3 in HRS cells of cHL. Streptavidin-biotin peroxidase-labeled (LSAB) immunohistochemical procedure: brown staining, magnification ×400; (**d**) histogram depicting the percentages of cHL cases in relation to the percentages of nuclear GATA3 immunohistochemical expression in HRS cells; (**e**) nuclear immunohistochemical expression of GATA3 in small lymphoid cells in the germinal center, the mantle zone, and the parafollicular zone in control reactive lymph node. Streptavidin-biotin peroxidase-labeled (LSAB) immunohistochemical procedure: brown staining, magnification ×200; (**f**) nuclear immunohistochemical expression of GATA3 in small lymphoid cells in the germinal center and the mantle zone of control reactive lymph node. Streptavidin-biotin peroxidase-labeled (LSAB) immunohistochemical procedure: brown staining, magnification ×400.

Nuclear immunohistochemical expression of GATA3 was found in HRS cells in 39/73 (54%) cases of cHL (Figure 1a–c). Both intensity and percent positivity of immunostaining varied greatly (the range of nuclear immunopositivity in HRS cells was 10–85%). The percentages of cHL cases in relation to the percentages of GATA3 positivity in HRS cells are depicted in the histogram (Figure 1d). In this histogram, 18.6%, 25.4%, and 10.2% of cHL exhibit 10–30%, 30–80%, and >80% GATA3 immunopositivity in HRS cells, respectively (Figure 1d). The median value of GATA3 expression was 10% and the mean value was 26%. NS subtype showed the highest positivity rate (35/56, 63%), followed by MC (4/14, 29%), and LR (0/3). GATA3 expression was not found in any of the four NLPHL immunostained cases.

We considered two thresholds for statistical analysis: a. any GATA3 positivity of HRS cells (at least 10%, which was the lowest GATA3 value in our series), and b. since the mean value was 26%, cases with expression of GATA3 ≥ 26% were considered as GATA 3 positive while cases with expression of GATA 3 < 26% were considered as GATA 3 negative. Using these two thresholds, no statistically significant correlation was found between GATA3 expression and the histological subtype of cHL. 

In the control normal lymph node, a few scattered small lymphocytes with rounded nuclei (moderate nuclear GATA3 immunostaining) and some spindle-shaped cells with elongated nuclei in the capsule (strong nuclear GATA3 immunostaining) were observed Figure 1e,f). The results in the reactive lymphoid tissue of the control tonsil were similar.

**Figure 2:**
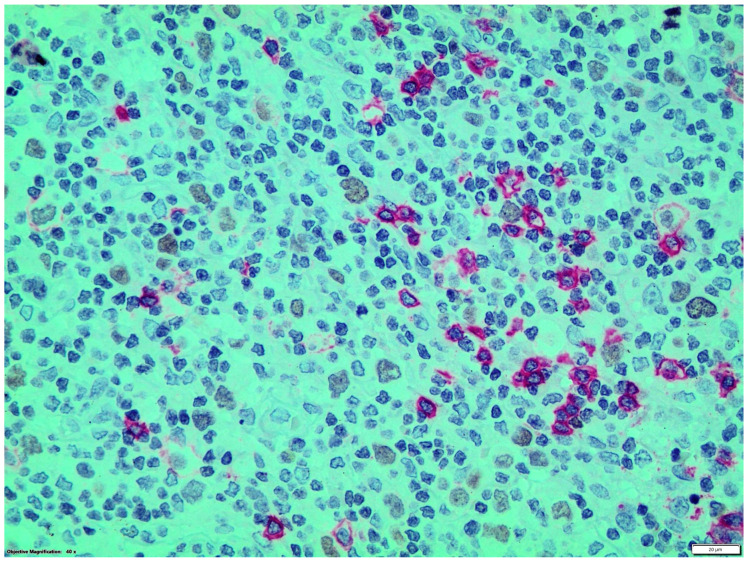
Double streptavidin-biotin peroxidase-labeled (LSAB) (brown staining)/alkaline phosphatase/anti-alkaline phosphatase (APAAP) (red staining) immunohistochemical procedure: CD20+ (red staining)/GATA3- B-cells and CD20-/GATA3+ (brown staining) HRS cells (magnification ×400).

**Figure 3:**
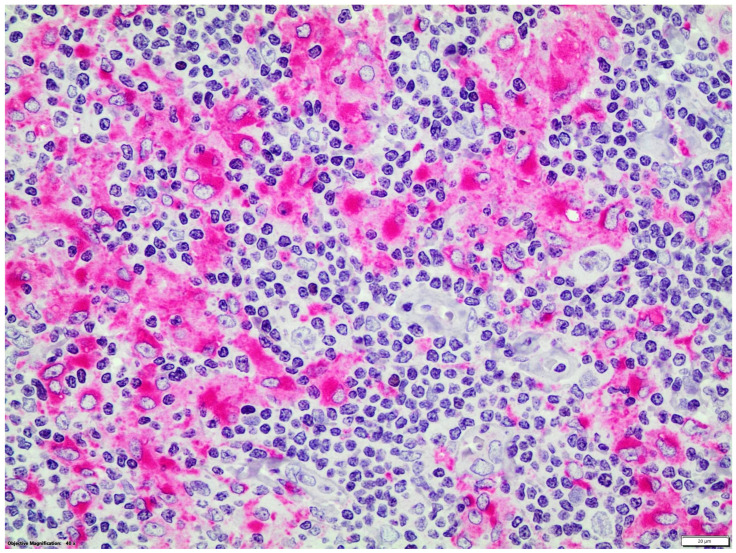
Double LSAB/APAAP immunohistochemical procedure: CD68+ (red staining)/GATA3- macrophages (magnification ×400).

Double immunostainings showed that nuclear GATA3 was expressed by some CD30+ or CD15+ HRS cells and by a few CD3+ T-cells which were frequently organized as small groups, distant from HRS cells. GATA3 nuclear expression was not detected in CD20, CD56 or CD68 + cells (Figure 2 and Figure 3). 

### 3.2. Correlation with Clinical and Laboratory Data 

Correlation of the GATA3 immunohistochemical results with clinical and laboratory data was possible in 59 patients with cHL, of whom 33 were males and 26 females. Of them, 32 cases exhibited expression of GATA3 in HRS cells (the range of positivity in HRS cells was 10–85%) while 27 cases had no detectable expression of GATA3 in HRS cells. The biological and clinical parameters of the patients at the time of cHL diagnosis as well as the parameters of interim PET status and progressive or primary refractory disease were analyzed in relation to the levels of GATA3 expression. The results are presented in Table 1 and Table 2.

The absence of GATA3 expression (considering the threshold of positivity as the mean value of GATA3 expression which was 26%) showed statistically significant correlation with the age of diagnosis as well as with β2-microglobulin (β2M) levels. Specifically, 30/38 (78.9%) of GATA3- patients were older than 45 years, in comparison with only 5/21 (23.8%) of GATA3+ patients (*p*: <0.001). A total of 9 out of 32 GATA3- patients had increased levels of serum β2M while none of GATA3+ patients had increased levels (*p* = 0.018). There was no correlation between GATA3 expression and the presence of anemia (hemoglobin < 10.5 g/dL), lymphopenia, leukocytosis (>15 × 10^9^/L), elevated levels of lactate dehydrogenase (LDH), elevated serum total bilirubin (TBIL), increased erythrocyte sedimentation rate (ESR) (>50 mm/h), hypoalbuminemia (albumin < 4 mg/dL), B-symptoms, extranodal disease, advanced stage in Lugano Classification, and bulky disease at the time of diagnosis of cHL. Moreover, positive interim PET and the presence of progressive or refractory disease were not associated with GATA3 expression. 

### 3.3. Survival Analysis

GATA3 expression had no effect on OS in univariate analysis. Univariate analysis revealed that increased levels of β2M and the presence of B-symptoms were significantly associated with inferior OS rates, while TBIL levels were of marginal significance (Table 3).

In a multivariate model evaluating GATA3 expression, increased β2M levels, presence of B-symptoms, and elevated TBIL levels, only β2M (*p* = 0.018) and B-symptoms (*p* = 0.035) had an independent effect on OS, while GATA3 expression had no effect on OS (*p* = 0.69) (Table 4).

### 3.4. EBV Status 

Expression of EBER1/2 transcripts was detected in variable proportions of malignant HRS cells in 19/62 studied cases of cHL (31%). The results for the 52 cases were reported previously (17). No statistically significant correlation between GATA3 expression patterns (positive vs negative cases) and the status of EBV (EBER1/2 positive vs EBER1/2 negative cases) was found. In particular, using 10% as a cut off for GATA3 expression, 9/20 (45%) of GATA3 negative cases were EBER1/2 positive, while 5/29 (17.2%) of GATA3 positive cases were EBER1/2 positive (*p* = 0.074). Using 26% (the mean value) as a cut off for GATA3 expression, 10/30 (33.3%) of GATA3 negative cases were EBER1/2 positive while 4/19 (21.1%) of GATA3 positive cases were EBER1/2 positive (*p* = 0.51).

## 4. Discussion

The present study, in agreement with previous reports, has demonstrated aberrant immunohistochemical expression of GATA3 in HRS cells in a substantial proportion of cHL [12,13,14,15]. The percentage of HRS cells with GATA3 nuclear immunohistochemical positivity in the present study was 54% (39/73 cases) and in previous studies the results were as follows: in Stanelle at al., 56% (9/16 cases) [12]; in Atayar et al., 67% (8/12 cases) [13]; in Kezlarian et al., 80% (34/49 cases) [14]; and in Kim et al., 76% (10/13 cases) [15]. The novel finding of the present study was the precise immunophenotypical characterization of the cell types expressing GATA3 using double immunohistochemistry in cHL tissues. We found that GATA3 was expressed by CD30+ or CD15+ HRS cells and a few CD3+ T-cells, whereas GATA3 expression was not detected in CD20+, CD56+ or CD68 + cells. These findings suggest that B-cells of the reactive microenvironment, in contrast to HRS cells which express an abortive B-cell differentiation program, do not express GATA3 which, as expected, is expressed by non-malignant T-cells.

Notably, Kezlarian et al. demonstrated that within the context of lymphoid malignancies with large Reed–Sternberg-like cells embedded in a reactive cellular milieu, GATA3 immunohistochemical expression is highly suggestive of cHL, and effectively excludes NLPHL with 100% negative predictive value (all 17 cases were negative) [14]. We confirmed that GATA3 is undetectable in NLPHL (all four cases studied were immunonegative). However, as 20–45% cHL can be immunonegative for GATA3, cHL cannot be excluded with negative GATA3 immunostaining [12,13,14,15].

Since constitutive activity of NF-kB and Notch pathways is a hallmark of HRS cells [20,21,22,23] and, as mentioned above, Stanelle et al. have linked GATA3 expression in HRS cells to the constitutive activity of NF-kB and Notch pathways, the GATA3 negativity in a sizeable portion of cHL cases requires further studies to be explained [12,13,14,15]. It could be hypothesized that GATA3 downregulated expression in HRS cells in a subset of cHL results from reduced NF-kB and/or Notch activity. This could be supported by the in vitro findings of Stanelle et al. who showed that decreased NF-κB activity resulted in variable levels of downregulated GATA3 expression in all four cHL cell lines; GATA3 expression was downregulated by approximately 1.5-fold in the L-428 and L-1236 cell lines and by 2.5- to 3-fold in the KM-H2 and U-HO1 cell lines [12]. Moreover, immunohistochemical studies reported heterogenous (absence, low or high) expression levels of members of the NF-kB family in HRS cells [24,25]. For example, Barth et al. found that 20/26 studied cases of cHL exhibited nuclear c-Rel immunostaining in variable proportions of HRS cells (the range of positivity was 30%–>70%) whereas the remaining 6 cases showed a complete absence of nuclear c-Rel expression in HRS cells [24]. Moreover, Nam-Cha et al. detected c-Rel and Rel-B nuclear immunostaining in HRS cells in 60% (35/58) and 54% (31/57) of cHL cases, respectively [25]. An alternative, but not mutually exclusive hypothesis to explain GATA3 negative cHL is that GATA3 expression in HRS cells in these cases is suppressed by alternative cellular pathways, thereby contributing to the biological heterogeneity of cHL. It would be of interest, in future studies, to analyze the immunohistochemical expression patterns of NF-kB and Notch proteins in HRS cells in relation to the GATA3 expression patterns (positive vs negative cases) to gain further insight into the potential biological and clinical significance of the heterogenous expression patterns of GATA3 in HRS cells in cHL.

Interestingly, a recent study showed that GATA-3 is a proto-oncogene in T-cell lymphoproliferative neoplasms including those derived from T-cell progenitors and their mature progeny [10]. On the other hand, GATA3 had no prosurvival effects in HL cell lines [12]. Indeed, Stanelle et al. investigated the effect of GATA3 knockdown on the induction of survival and apoptotic cell death in various HL cell lines [12]. They used an MTT assay to measure the cell viability and immunoblot analysis for detection of cleaved caspase-3 which is a marker for apoptotic cells, but they did not detect clear evidence of caspase-dependent apoptotic cell death in any of the three cHL cell lines analyzed (L-428, L-1236 and KM-H2) [12]. On the basis of the above findings, Stanelle et al. concluded that GATA3 does not influence the survival of HL cells in their study models [12]. Thus, further studies are needed to investigate the putative oncogenic potential of GATA3 in a portion of cHL. One hypothesis could link GATA3 with the oncogenic potential of EBV since GATA3 was expressed in all but one GC-lymphoblastoid B-cell lines (LCL) [16], indicating that EBV-infection can induce deregulation of expression of transcription factors that are involved in cell lineage decisions. Activation of GATA3 in GC-LCL may be partly due to EBNA2 expression, as EBV-encoded EBNA2 function is similar to an activated Notch1 receptor, which has been described to activate GATA3 [24,25]. However, in the present study, we found no statistically significant correlation between GATA3 expression and EBV status. With respect to the clinical impact of GATA3 immunopositivity in cHL, a previous study reported that the GATA3+ cHL group was significantly associated with an increase in the number of lymph node sites compared to GATA3- cHL (*p* = 0.007) (taking 10% as the threshold of positivity, which was the lowest value) [14]. In our study, the GATA3- cHL group (taking 26% as the threshold of positivity, which was the GATA3 mean value) showed a statistically significant correlation with unfavorable prognostic factors such as older age at diagnosis and increased levels of serum β2-M [26,27,28,29,30,31,32]. Although the findings of the present study have no major clinical relevance now, they are of interest regarding the biology of cHL, since they provide further evidence supporting the biological heterogeneity of cHL. Larger and prospective clinico-pathological studies are needed to better delineate what clinical implications GATA3 expression in HRS cells might have.

## 5. Conclusions

In conclusion, heterogenous nuclear expression of GATA3 in HRS cells was observed in a substantial proportion of cHL cases with the NS subtype showing the highest positivity rate. These findings further support the heterogeneous biological features of cHL. Furthermore, the GATA3 negative cHL group showed statistically significant correlation with unfavorable prognostic factors, suggesting that further studies are needed to analyze the prognostic impact of GATA3 expression in cHL.

## Figures and Tables

**Table 1:** Clinical and biological characteristics of patients with classical Hodgkin lymphoma in relation to the expression of GATA 3 (cut-off 10%). Statistically significant associations are marked in bold. ESR: erythrocyte sedimentation rate, LDH: lactate dehydrogenase, PET/CT: positron emission tomography/computed tomography.

Parameter	No GATA3 Expression	GATA3 Expression	*p*-Value (χ^2^ or Fisher Exact Test)
Age of diagnosis > 45 years old	21/27 (77.8%)	14/32 (43.8%)	**0.017**
Hemoglobin < 10.5 g/dL	3/27(11.1%)	8/32 (25%)	0.2
Lymphopenia (<0.6 × 10^9^/L)	2/27 (7.4%)	3/32 (9.4%)	1.0
White Blood Cells > 15 × 10^9^/L	3/27 (11.1%)	4/32 (12.5%)	1.0
Increased LDH	12/26 (46.2%)	18/31 (54.4%)	0.36
Albumin < 4 mg/dL	14/26 (53.8%)	18/31 (58.1%)	0.74
β2-microglobulin increased	6/21 (28.6%)	3/30 (10%)	0.14
Male	16/27(59.3%)	17/32 (53.1%)	0.83
B symptoms	12/27 (44.4%)	18/32 (56.3%)	0.52
Lugano stage advanced	18/27 (66.7%)	19/32 (59.4%)	0.56
Bulky disease	4/27 (14.8%)	7/32 (21.9%)	0.52
Positive interim PET/CT	2/7 (28.7%)	7/19 (36.8%)	1.0
Progressive disease/primary refractory	5/25 (20%)	13/31 (41.9%)	0.14
ESR > 50 mm/h	12/26 (46.2%)	13/31 (41.9%)	0.75
Extranodal disease	10/27 (37%)	8/32 (25%)	0.31

Statistically significant associations are marked in bold.

**Table 2:** Clinical and biological characteristics of patients with classical Hodgkin lymphoma in relation to the expression of GATA 3 (cut-off: mean value 26%). Statistically significant associations are marked in bold. ESR: erythrocyte sedimentation rate, LDH: lactate dehydrogenase, PET/CT: positron emission tomography/computed tomography, TBIL: total bilirubin.

Parameter	No GATA3 Expression	GATA3 Expression >26%	*p*-Value (χ^2^ or Fisher Exact Test)
Age of diagnosis > 45 years old	30/38 (78.9%)	5/21 (23.8%)	**<0.001**
Hemoglobin < 10.5 g/dL	6/38(15.8%)	5/21 (23.8%)	1.0
Lymphopenia (<0.6 × 10^9^/L)	3/38 (7.9%)	2/21 (9.5%)	1.0
White Blood Cells > 15 × 10^9^/L	6/38 (15.8%)	1/21 (5%)	0.4
Increased LDH	16/37 (43.2%)	14/20 (70%)	0.098
Albumin < 4 mg/dL	21/37 (56.8%)	11/20 (55%)	1.0
β2-microglobulin increased	9/32 (28.1%)	0/19 (0%)	**0.018**
Male	21/38(55.3%)	12/21 (57.1%)	1.0
B symptoms	17/38 (44.7%)	13/21 (61.9%)	0.32
Lugano stage advanced	25/39 (64.1%)	12/20 (60%)	0.98
Bulky disease	6/38 (15.8%)	5/21 (23.8%)	0.68
Positive interim PET/CT	4/10 (40%)	5/16 (31.3%)	0.69
Progressive disease/primary refractory	8/35 (22.9%)	10/21 (47.6%)	0.1
ESR > 50 mm/h	17/37 (43.2%)	9/20 (45%)	1.0
Extranodal disease	12/38 (31.6%)	6/21 (28.6%)	1.0
TBIL increased	7/38 (18.4%)	0/20 (0%)	0.08

Statistically significant associations are marked in bold.

**Table 3:** Univariate analysis. Statistically significant associations are marked in bold. ESR: erythrocyte sedimentation rate, LDH: lactate dehydrogenase, PET/CT: positron emission tomography/computed tomography, TBIL: total bilirubin, OS: overall survival.

Characteristic	5 Year-OS (%)	*p*-Value
GATA 3 (−)	79%	0.99
GATA 3 (+)	77%	
GATA 3 < 26%	79%	0.97
GATA 3 ≥ 26%	60%	
Age of diagnosis ≤ 45 years old	77%	0.18
Age of diagnosis > 45 years old	72%	
Hb ≥ 10.5 g/dL	79%	0.13
Hb < 10.5 g/dL	53%	
Lymphopenia (≥0.6 × 10^9^/L)	75%	0.66
Lymphopenia (<0.6 × 10^9^/L)	75%	
Female	83%	0.4
Male	67%	
White Blood Cells ≤ 15 × 10^9^/L	73%	0.58
White Blood Cells > 15 × 10^9^/L	85%	
Normal LDH	72%	0.85
Increased LDH	76%	
Albumin ≥ 4 mg/dL	82%	0.12
Albumin < 4 mg/dL	67%	
β2-microglobulin normal	83%	**<0.001**
β2-microglobulin increased	27%	
No B symptoms	90%	**0.008**
B symptoms	59%	
Lugano stage limited	82%	0.18
Lugano stage advanced	71%	
No Bulky disease	73%	0.56
Bulky disease	83%	
Negative interim PET/CT	65%	0.2
Positive interim PET/CT	47%	
No Progressive/primary refractory disease	85%	0.19
Progressive disease/primary refractory	61%	
ESR≤ 50 mm/h	72%	0.76
ESR > 50 mm/h	77%	
No Extranodal disease	70%	0.35
Extranodal disease	87%	
TBIL normal	77%	0.05
TBIL elevated	54%	

Statistically significant associations are marked in bold.

**Table 4:** Cox regression analysis. Multivariate analysis. Statistically significant associations are marked in bold. TBIL: total bilirubin.

Characteristic	Hazard Ratio	95% Cl	*p*-Value
β2-microglobulin increased	14.6	1.6–133.6	**0.018**
B symptoms	9.8	1.18–83.2	**0.035**
TBIL increased	1.2	0.21–7.29	0.81
GATA3 ≥ 26%	1.45	0.23–8.9	0.69

Statistically significant associations are marked in bold.

## Data Availability

Research data are available upon request.
